# Fifty-year change in air pollution in Kaohsiung, Taiwan

**DOI:** 10.1007/s11356-022-21756-z

**Published:** 2022-07-04

**Authors:** Chiu-Hsuan Lee, Peter Brimblecombe, Chon-Lin Lee

**Affiliations:** 1 Department of Marine Environment and Engineering, National Sun Yat-sen University, Kaohsiung, Taiwan; 2 Aerosol Science and Research Center, National Sun Yat-sen University, Kaohsiung, Taiwan; 3 Department of Public Health, Kaohsiung Medical University, Kaohsiung, Taiwan; 4 Department of Applied Chemistry, Providence University, Taichung, Taiwan

**Keywords:** Aerosols, Economic change, Health effects, Agricultural effects, Visibility

## Abstract

The change in air quality in cities can be the product of regulation and emissions. Regulations require enforcement of emission reduction, but it is often shifting economic and societal structures that influence pollutant emissions. This study examines the long-term record of air pollutants in Kaohsiung, where post-war industrialisation increased pollution substantially, although improvements are observed in recent decades as the city moved to a more mixed economy. The study tracks both gases and particles across a period of significant change in pollution sources in the city. Concentrations of SO_2_ and aerosol SO_4_^2−^ were especially high ~1970, but these gradually declined, although SO_4_^2−^ to a lesser extent than its precursor, SO_2_. While twenty-first century emissions of SO_2_ and NO_x_ have declined, this has been less so for NH_3_, because it arises from predominantly agricultural sources. The atmosphere in Kaohsiung continues to have high concentrations of O_3_, and these have risen in the city, likely a product of less titration by NO. The changes have meant that ozone has become an increasing threat to health and agriculture. Despite a potential for producing (NH_4_)_2_SO_4_ and NH_4_NO_3_ aerosols, a product of a relatively constant supply of NH_3_, visibility has improved in recent years. Emissions of SO_2_ and NO_x_ should continue to be reduced, as these strongly affect the amount of fine secondary aerosol. However, the key problem may be ozone, which is difficult to control as it requires careful consideration of the balance of NO_x_ and hydrocarbons so important to its production.

**Supplementary Information:**

The online version contains supplementary material available at 10.1007/s11356-022-21756-z.

## Introduction

Air quality in many cities has improved in line with changes in their economy and the regulation of emissions. Declining concentrations of air pollutants, most notably SO_2_, but later NO_x_, can be ascribed to changes in industries and their control, and a more modern vehicle fleet (Brimblecombe 2005; Power and Worsley 2018). However, the link between emission control and reduced air pollutant concentrations is weakened because of a mediating atmospheric chemistry, best characterised by O_3_ formation. Its production is affected by hydrocarbons and nitrogen oxides. Ozone concentrations can increase when nitrogen oxide emissions decrease, so air pollution regulation needs to go beyond simple emission control and requires the application of air quality management (Elsom 1992). Particulate matter is an important contributor to air pollution, yet a significant fraction of the urban aerosol is again produced through reactions of primary pollutants in the atmosphere that lead to both inorganic components (Ravishankara 1997), such as sulphates and nitrates. Secondary sulphate aerosol was probably more abundant in the past when SO_2_ levels were high. Secondary organic components were best represented by the carboxylic acids, perhaps most notably low volatility dicarboxylic acids (Kawamura et al. 2001).

**Fig. 1:**
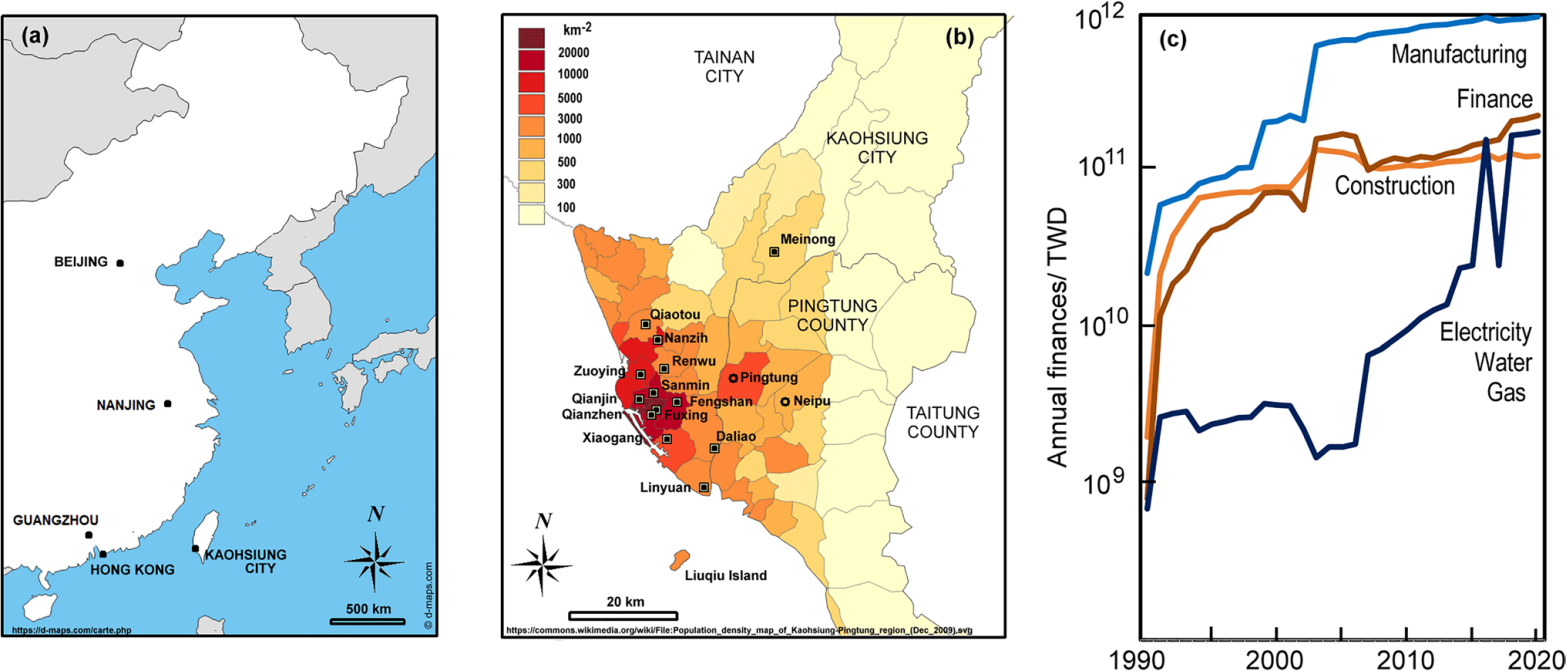
**a** Regional and **b** local maps showing main places mentioned in the text within the Kaohsiung City special municipality and neighbouring Pingtung County, where ammonia was sampled. The large squares mark the Environmental Protection Administration sites mentioned in the text. Shading reveals the 2009 population density. **c** Financial capital for the various economic sectors in Kaohsiung

Taiwan (Fig. 1) experienced a rapid transformation from the large agrarian colony left by the Japanese after World War 2, when it saw innovative expansion, such as that in the semiconductor industry. Taiwan’s industrial growth saw per-capita gross domestic product in US dollar increase from $397 to $8200 by 1990 and just over $28000 by 2020. Kaohsiung could share in this as during the colonial period, its harbour became a focus for shipping and rail transport that allowed the city’s development as a major hub for Taiwan’s south, with an industrial base in steel, cement, petrochemicals, paper making, etc. The Taiwan Economic Miracle (Tsai 1999) saw rapid growth of industrial infrastructure (~1960–1990), which contributed to pollutant emissions that, in the early pre-regulated stages, paralleled the strengthening economy. Today, Kaohsiung remains an industrial city, though with an increasing shift in the local economy (Fig. 1c) towards financial services, tourism, and the arts, with plans for the waterfront to become a landscape resource (KEC 2021).

Post war industrialisation led to regulations needed to improve air quality, initially under the *Air Pollution Control Act* of 1975. The relaxation of Martial Law (late 1980s) saw newly democratised systems, and though electoral politics can stifle environmental debate, Taiwan established a cabinet-level Environmental Protection Administration (TEPA) in 1987. However, the 1975 *Act* was only effective after 1992, when stricter rules were implemented (Tang 1993). Under democratisation, this “credit for the improvement has been given to the air emission fee program that was first implemented” in 1995 (Tang and Tang 2000). Before then, “the traditional command-and-control program and tax-allowance subsidy were the two major instruments used for air pollution control …” (Shaw and Hung 2001). Emission standards were established for power facilities 1994-05-04, while 1995-07-01 saw the introduction of an air pollution control fee for SO_x_ emissions, and subsequent regulations to reduce volatile organic emissions (see supplementary table in Chen et al. 2014).

Post-war Kaohsiung expanded with an urban population of 168,008 in 1947 to 1,512,798 in 2017, where there were 2,776,912 in the municipal area. In parallel, there was a growth of heavy industry (1976–1986) and a mature stage for the heavy chemical industry (1986–1996). Industrial zones were established in Fengshan (1974), Yongan, and Linyuan (1974–1975). Although the service industry has surpassed manufacturing in terms of value, steel, petrochemical, cement, shipbreaking, and processing, exports remain substantial, though characterised by pollution. *Challenge 2008: National Development Key Project* encouraged investments in infrastructure such as High Speed Rail, Kaohsiung MRT (Mass Rapid Transit), but with newer less polluting developments emerging from 2009: (i) biotechnology, (ii) tourism, (iii) green energy, (iv) medical care, (v) low intensity agriculture, and (vi) cultural creativity (KCG 2010).

The long history of industrial emissions has promoted many studies of air pollution in Kaohsiung, with deposit gauge measurements from the 1960s (Hsu and Wei 1971; Selya 1975; Wei 1966), and more modern measurements from the 1970s (Chow et al. 1983). In Europe and North America, there are estimates of long-term air pollutant loads in urban air using modelling (Brimblecombe 1977), observations of smoke days (Davidson 1979) or pollutant deposition (Brimblecombe 1982), but fewer from Asia (Ishikawa and Hara 1997). Taiwan’s National Health Administration planned an optimal monitoring network for SO_2_ in Kaohsiung from 1975 to 1977 (Liang and Lee 1980). There is a long series of studies of health (Yang et al. 1998), visibility (Lee et al. 2005; Lee 2006), and PAHs (Lai et al. 2017). Early problems seem dominated by primary pollutants, but the situation with ozone has caused concern across recent years (Hung-Lung et al. 2007; Shiu et al. 2007; Tsai et al. 2012). Although there are also contemporary claims that for two decades, atmospheric visibility in Kaohsiung has worsened (Lee and Lai 2018); they are not supported by Maurer et al. (2019), who suggest 2000–2015 saw improvement in Kaohsiung, paralleling change elsewhere in Taiwan, but not fully explained by lower RH or PM_10_.

Ammonia has not often been measured in Taiwan, but it may be important, given the density of pigs and poultry (Cheng et al. 2011). Additionally, Hsieh and Chen (2010) measured NH_3_ at industrial parks in southern Taiwan at Neipu, Pingtung, and Pingnan over two consecutive days each (2003-09/2004-12), finding means of 90.4 ppb, 72.8 ppb, and 84.9 ppb, while at the National Pingtung University of Science and Technology campus dormitory and a bamboo grove near the village of Laopi in Pingtung County mixing ratios were 52.2 ppb and 4.6 ppb showing the significance in the region. Vehicles represent a potential source of NH_3_ in urban areas; e.g. in urban Guangzhou, vehicles produce 19% of the ammonia (Liu et al. 2014), although 2006 estimates across the Pearl River Delta suggest vehicles accounted for just 2.5% (Zheng et al. 2012).

This paper explores a 50-year history of air pollution in Kaohsiung to understand the changes and assess the relevance of shifts in the economy and regulatory activity. The city represents an interesting and somewhat isolated location compared to the Greater Taipei Area in the north. Additionally, some pollutants are transported across the Taiwan Strait from Mainland China, but that has limited impact on urban concentrations (Lai and Brimblecombe 2021). In Kaohsiung, there have been great changes as it moved from an unregulated industrial centre for manufacturing, steelmaking, oil refining, and shipbuilding to a place aspiring to be noted for international exhibitions, tourism, and the arts. This transition has required more stringent urban planning and concerns over air quality and visibility. Although coastal cities have been well studied, the difficulty of maintaining sites may limit measurement duration (e.g. Alastuey et al. 2004; Galindo et al. 2020), but the longer record used here means we can explore how urban transformation is reflected through a half century of development. We give special attention to changes in the threat to health, agricultural production, and visibility.

## Method

### Economic, air pollution, and meteorological data

**Fig. 2:**
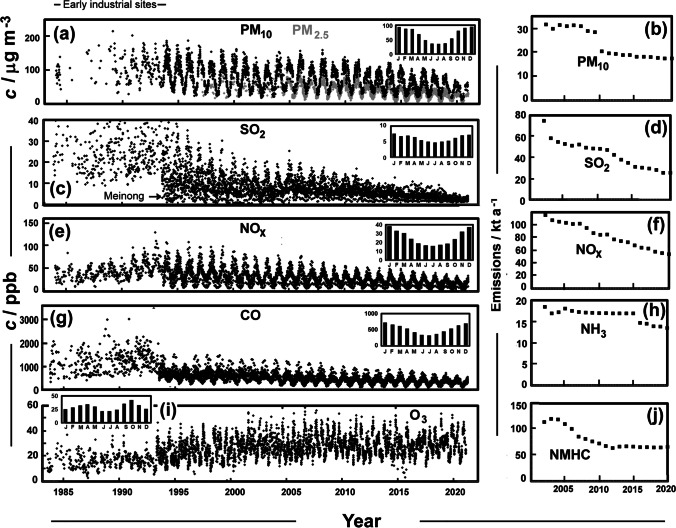
Monthly average TEPA measurements from sites in Kaohsiung, with seasonal patterns (averaged 2000–2020) as insets and annual emissions from the Kaohsiung special municipality. (**a**) PM_10_ (black) and PM_2.5_ (grey) concentrations, (**b**) PM_10_ emissions, (**c**) SO_2_ mixing ratios (rural Meinong noted as low concentration series), (**d**) SO_2_ emissions, (**e**) NO_x_ concentrations, (**f**) NO_x_ emissions, (**g**) CO concentrations, (**h**) NH_3_ emissions, (**i**) O_3_ concentrations, (**j**) NMHC emissions

The project used economic data from the Kaohsiung City Government, Department of Budget, Accounting and Statistics as plotted in Fig. 1c (KCG 2021; KCGDG 2021) and population data (DHR 2021). Energy use has grown since 1970 with about 4 TWh and 7.5 TWh from oil and coal to level values of around 700 TWh and 400 TWh for these fuels from 2000 (BP 2021). Gas has become more important and now amounts to 200 TWh (BP 2021). A network of sites (Taiwan Air Quality Monitoring Network, TAQMN) is maintained by the TEPA to measure air pollutants (https://airtw.epa.gov.tw). In Kaohsiung, it began with sites at Sanmin, Fengshan, Fuxing, and Qianjin providing data, though incomplete, from 1984. Nanzih came 2 years later, and a widening network added observations from Qianzhen, Daliao, Renwu, Xiaogang, Meinong, Linyuan, Qiaotou, and Zuoying from 1993 onwards (Fig. 1). Early monitoring in Kaohsiung was weighted towards crowded industrial areas of the city, but became more widespread over time, with a site placed at Meinong, a Hakka farming community on the Laonong River, 40 km from the centre of Kaohsiung. Corrections to PM_2.5_ from 2014 adopted the USEPA Non-Federal Reference Method, which led to a reduction in average values (~25%), but has little effect on our work as we largely avoid using the fine particulate data. Emission estimates (https://teds.epa.gov.tw/) are tuned to meet the boundaries of current Kaohsiung City, now a county-sized special municipality (area ~2950 km^2^). These along with concentration and mixing ratios (*c*) are plotted in Fig. 2. Aerosol composition is less frequently measured in the region, and much has been done as a part of research projects, rather than regular monitoring, though the most relevant data is given in the supplement. Daily visibility data for Kaohsiung (WMO_ID:467440) were extracted as daily observations from the historical record (https://e-service.cwb.gov.tw/HistoryDataQuery/index.jsp) on the CODiS of Taiwan’s Central Weather Bureau. The records displayed in Fig. 2 come from a single source, so the methodology remains consistent, except for a change to the TEPA methodology for PM_10_ in 2010 as seen in emissions in Fig. 2(b). Additionally, there was a decade-long break to the ozone record for Fuxing.

The number of data points was often large, so we used parametric methods (e.g. Welch’s *t*-test), but where small and the distribution undefined, non-parametric techniques were preferred along with the median and quartile ranges. The Wilcoxon signed-rank test (rather than a *t*-test) was used where the data set was small and occasionally Kendall *τ* and Theil-Sen slopes were determined as these are more robust against outliers than a classical linear regression (Vannest et al. 2016).

## Results and discussion

The record of pollutants PM_10_ PM_2.5_, SO_2_, NO_x_, NO_2_, and O_3_ as measured by TAQMN are shown in Fig. 2.

### Decadal change in primary pollutants

Monthly average pollutant concentrations and mixing ratios from the TAQMN site in Kaohsiung and estimated emissions for the region 2002–2020 are shown in Fig. 2. There are distinct annual cycles to the primary pollutants (Fig. 2(a, c, e, g)), higher values occurring each winter (Lee et al. 2018; Tsai et al. 2013); seasonal cycles 2000–2020 appear as insets. Trends across this period suggest continuous improvement to the primary pollutants such as NO_x_ and PM_10_ (*τ*=−0.40, *p*<.0001 and *τ* =−0.23, *p*<.0001), and notably for SO_2_ (*τ*=−0.62, *p*<.000). Especially low SO_2_ values are evident at the rural Meinong site (Fig. 2(c)). There is evidence of a weaker mid-cycle in annual cycle for NO_x_ in rural areas (Fig. 2(e)). The SO_2_ mixing ratios were especially high before 1994, when measurements were made at crowded urban locations: Sanmin, Fengshan, Fuixing, Qianjin, and Nanzih. The mixing ratios typically continued to be higher than at other sites that entered the record after 1994. However, even at these crowded sites, levels declined over time, continuing improvement perhaps a result of sulphur emission fees beginning in 1995.

The mixing ratios of CO, NO_x_, and less clearly PM_10_ rose at first, but these decreased from the early 1990s, in a way typical of the changing pollutant levels during the historic development of cities (Brimblecombe 1977). By contrast, oxidants have increased (Chen et al. 2014), with O_3_ mixing ratios on the rise (Fig. 2(i)). In the eastern parts of Kaohsiung, toluene from paint and solvent industries plays an important role in O_3_ production as in inland areas production is often limited by the NMHCs, i.e. volatile organic compounds (Hung-Lung et al. 2007). Ozone production in the air aloft, often reflecting long-range transport, can be NO_x_ limited (Hung-Lung et al. 2007). The seasonal cycle of ozone, with a bimodal structure, is more complex than the primary pollutants (inset of Fig. 2(i)).

Estimated emissions from the Kaohsiung area for a range of pollutants from 2002 onward (Fig. 2(b, d, f, h, j)) reflect emission reduction policies (Chen et al. 2014). Emission inventories are error prone, with a factor of two errors possible for NO_x_ and hydrocarbons and an even larger threefold error found for CO and particulate matter (Smit et al. 2010), but when a consistent methodology is applied year by year trends can nevertheless be clear. However, the sudden change in PM_10_ in 2010 relates to an altered assessment methodology for industrial emissions, adopted by the TEPA*.* Some 24 kt a^−1^ ammonia was emitted from poultry farms in Taiwan (Cheng et al. 2011), which makes Kaohsiung’s estimated emissions substantial at 18.74 kt a^−1^ in 2002.

The decline in emissions appears to be smaller than that for concentration. This anomaly may arise because most pollutant concentration measurements are made in the built up and increasing residential area of Kaohsiung, while the emissions are for the county-sized special municipality.

Particulate matter has been measured for many years. Selya (1975) listed the 2-year average for total suspended matter as 371 μg m^−3^ and SO_4_^2−^ at 21.4 μg m^−3^. Although this early SO_4_^2−^ concentration is high, it seems compatible with later measurements for 1994/1995, 11.5 μg m^−3^ (Yang et al. 1998), and 1998/1999, 14.34±5.10 μg m^−3^ (Lin 2002), as tabulated in the supplement. It is supported by the trends in SO_2_ mixing ratios in the early part of the record (Fig. 2(c)) and the suggestions of high levels from Chow et al. (1983).

**Fig. 3:**
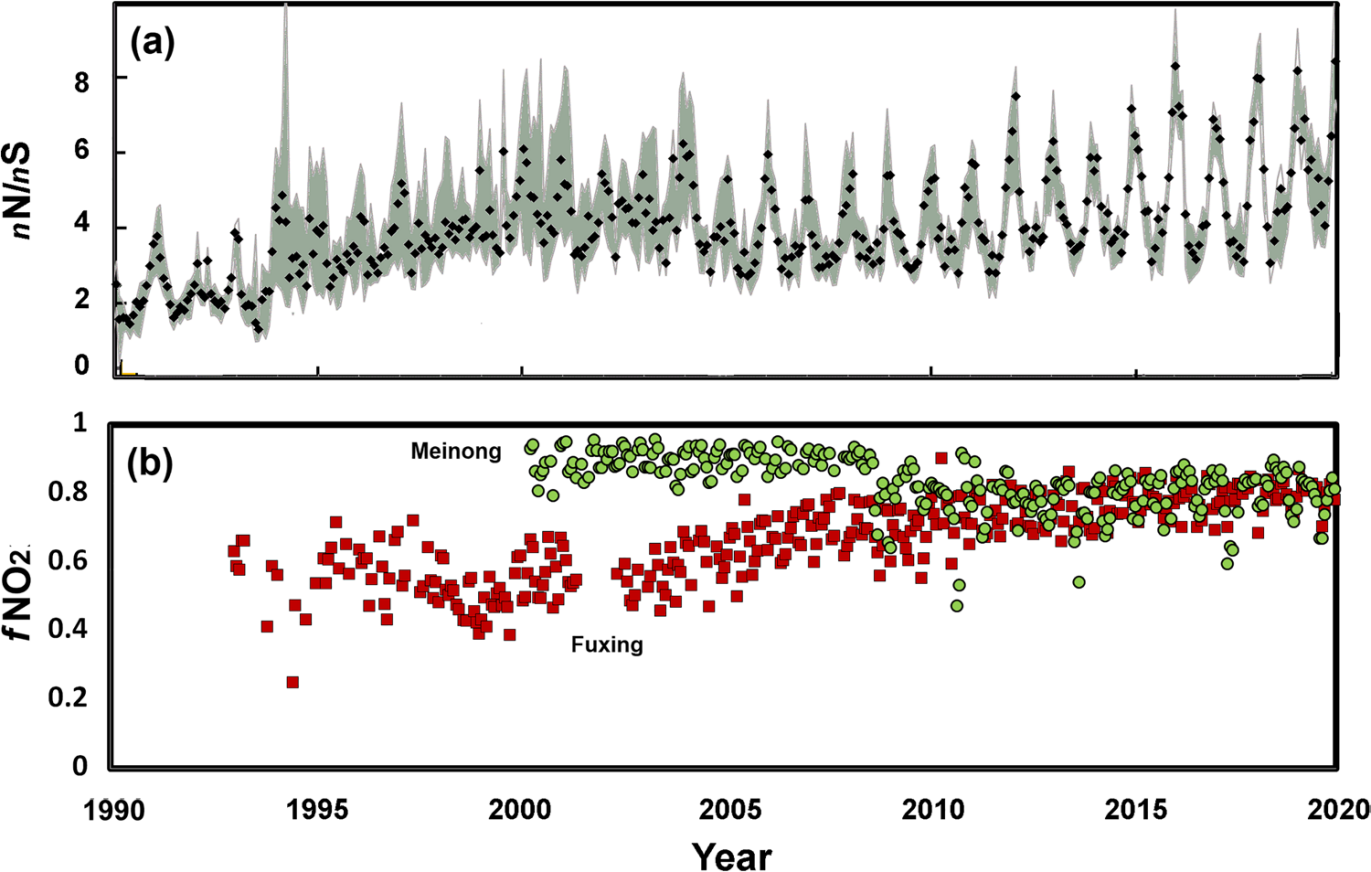
(**a**) Monthly average ratios of gaseous nitrogen (NO_x_) to sulphur (SO_2_) from TEPA measurements at sites in Kaohsiung. (**b**) The fraction of NO_x_ as NO_2_ (*f*_NO2_= NO_2_/NO_X_) at Fuxing (red squares) and Meinong (green circles)

Figure 3(a) shows the mole ratio of nitrogen to sulphur oxides in the gas phase (i.e. *n*_NOx_/*n*_SO2_ as points and a shaded interquartile range). Measurements from the late 1960s (Selya 1975) would suggest that in the particulate phase, *n*_NO3_/*n*_SO4_ (~0.24 in the 1960s) was lower than that at present in a sulphur-dominated atmosphere with uncontrolled industrial use of soft coal, a cheap fuel widely used in factories, hotels, dwellings, schools, etc. Such low values could have reflected large amounts of sulphate present in coarse fly ash. From the late 1960s, soft coal was banned in Taipei, so some entrepreneurs in Kaohsiung may have for a short time increased its use (Selya 1975). Such changes are attributed to the shift from coal to petroleum, and in Kaohsiung are reflected in the change in *n*_NOx_/*n*_SO2_ from 1.5 in the 1980s to 4.5 over the last decade.

Overall, these observations of long-term change in air pollutants in Kaohsiung show a pattern among primary pollutants similar to other cities along with social change that has shifted fuel use (e.g. London in Brimblecombe 2006). Pressure for regulation in Taiwan led to reductions in mixing ratios of SO_2_ first, with NOx and PM seeming to reach a maximum in the last decade of the twentieth century. However, as substantial as many of the improvements have been, the changing economy of the city has made a significant contribution to reductions. The NO_3_^−^/SO_4_^2−^ ratio was probably low in the 1970s and grew after that. The NO_x_/SO_2_ ratio in the atmosphere of Kaohsiung has increased, which follows early reduction of sulphur emissions, and enhanced by NO_x_ from a growing vehicle fleet that has been difficult to keep in check. In Taiwan, vehicle registrations are increasing at 0.24 million a year (https://tradingeconomics.com/taiwan/car-registrations), but there are additionally 0.9 million polluting motor-scooters (Everington 2018). Despite this, the overall mixing ratios on NO_x_ have declined in line with emissions (Fig. 2(e, f)) suggesting some regulatory success in responding to an enlarged automobile fleet, i.e. private vehicles from 1994, 432,228, to 2020, 763,975 (KCGDG 2021).

### Change in secondary pollutants

The changing volatile organic components as non-methane hydrocarbons (Fig. 2(j)) and the increasing dominance of NO_x_ (Fig. 3(a)) encourage the formation of secondary pollutants. Toluene has been shown to be particularly relevant to the formation of ozone (Hung-Lung et al. 2007), although Kuo et al. (2015) indicated that VOC was not significantly correlated with ozone variability in the few episodes studied in Kaohsiung. From 1994 to 2003, Shui et al. 227 (2007) found that the mixing ratios of NO_2_ in southern Taiwan decreased while those of ozone increased, which could be accounted for by (i) the reduction in NO_2_, due to lower NO titration, or (ii) the more reactive precursor NMHCs (Chang et al. 2005). Figure 3(b) shows the increasing fraction (*f*_NO2_= NO_2_/NO_x_) of NO_x_ present as NO_2_ at urban Fuxing and in rural Meinong. Despite being in an urban area, Fuxing has become increasingly less industrial over the decades, now focussed on commercial and residential activities. Over time, decreasing amounts of NO_x_ have allowed the available O_3_ to oxidise larger fractions of NO to NO_2_. A quarter century back, the 5-year average O_3_ at the urban site of Xiaogang was 19.9 ± 6.9 ppb (1993-08/1998-07), but much higher at rural Meinong 29.4 ± 6.6 ppb. More recently (2016-01/2020-12), the differences had narrowed to 26.4 ± 7.7 ppb and 27.4 ± 6.2 ppb. The increases in urban areas are typical of Southern China where titration of O_3_ by NO has decreased with declining emissions of NO_x_ (Li et al. 2022a).

These changes are likely accompanied by the formation of secondary inorganic aerosols that have been easy to trace in Hong Kong as the record of aerosol is detailed since 1995 (Brimblecombe 2022). The record of aerosol composition is less complete in Kaohsiung and fails to reveal a satisfying and coherent picture of change (see supplement Fig. S2), although it is likely that in the 1970s the sulphate was high. The Kaohsiung special municipality is agricultural, so the hinterland provides NH_3_ to neutralise acidity and these emissions have changed only a little over time (Fig. 2(h)). Aerosol NH_4_^+^ is probably insensitive to the total NH_3_, but highly sensitive to total H_2_SO_4_ and HNO_3_ (Cheng and Wang-Li 2019). Nevertheless, the special municipality has not been able to greatly reduce its agricultural NH_3_, but it is probably more critical to ensure that emissions of SO_2_ and NO_x_ continue to be reduced as these strongly affect the amount of fine secondary aerosol.

Chemical transformations mean that particulate SO_4_^2−^ and NO_3_^−^ concentrations might not necessarily follow regulatory improvements to their precursors. However, in Kaohsiung, it is likely that over longer timescales, particulate sulphate has declined in parallel with SO_2_. The Theil-Sen slope for the medians of the SO_4_^2−^ suggests a decline of ~0.3 μg m^−3^ a^−1^ from 1970, which would accumulate to ~80% over time. Since the mid-1990s, it decreased from 11.5–14.3 μg m^−3^ (Lin 2002; Yang et al. 1998) to 3.9–4.4 μg m^−3^ at present (Shen et al. 2020), i.e. ~65% decline. This is proportionally less than the decrease in SO_2_, from ~25 μg m^−3^ in the 1990s to ~3 μg m^−3^ at present (~90% decrease).

### Changing health risk

Air pollution poses both long- and short-term health risks. The risk of daily hospital admissions due to air pollution can be calculated based on exposure to pollutants, and here we adopted the method used to calculate the Air Quality Health Index of Hong Kong (GovHK, 2014). The risk is determined as the sum of percentage added health risk (*R*_AHR_) for daily hospital admissions attributable to the 3-h moving average mixing ratios of NO_2_, SO_2_, O_3_, and particulate matter (here taken as PM_10_). These risk factors were derived from health statistics and air pollution data from Hong Kong and are therefore not exact for Kaohsiung, but given similar population activity and climate, there should be a reasonable proportionality. The *R*_AHR,i_ for each pollutant *i*, as

1 $${R}_{\mathrm{AHR},\mathrm{i}}=100\left[\exp \left({\beta}_i{c}_i\right)-1\right]$$

and *c*_*i*_ is the 3-h moving average concentration of pollutants (μg m^−3^), with the factors *β*_NO2_ = 0.0004462559, *β*_SO2_ = 0.0001393235, *β*_O3_ = 0.0005116328, and *β*_PM10_ = 0.0002821751 (Wong et al. 2012). Although it would make more sense to use PM_2.5_ in these calculations, PM_10_ was used as the record was more complete, but PM_10_ can provide a reasonable estimate of the health risk, and it includes PM_2.5_ (Brimblecombe 2021).

**Fig. 4:**
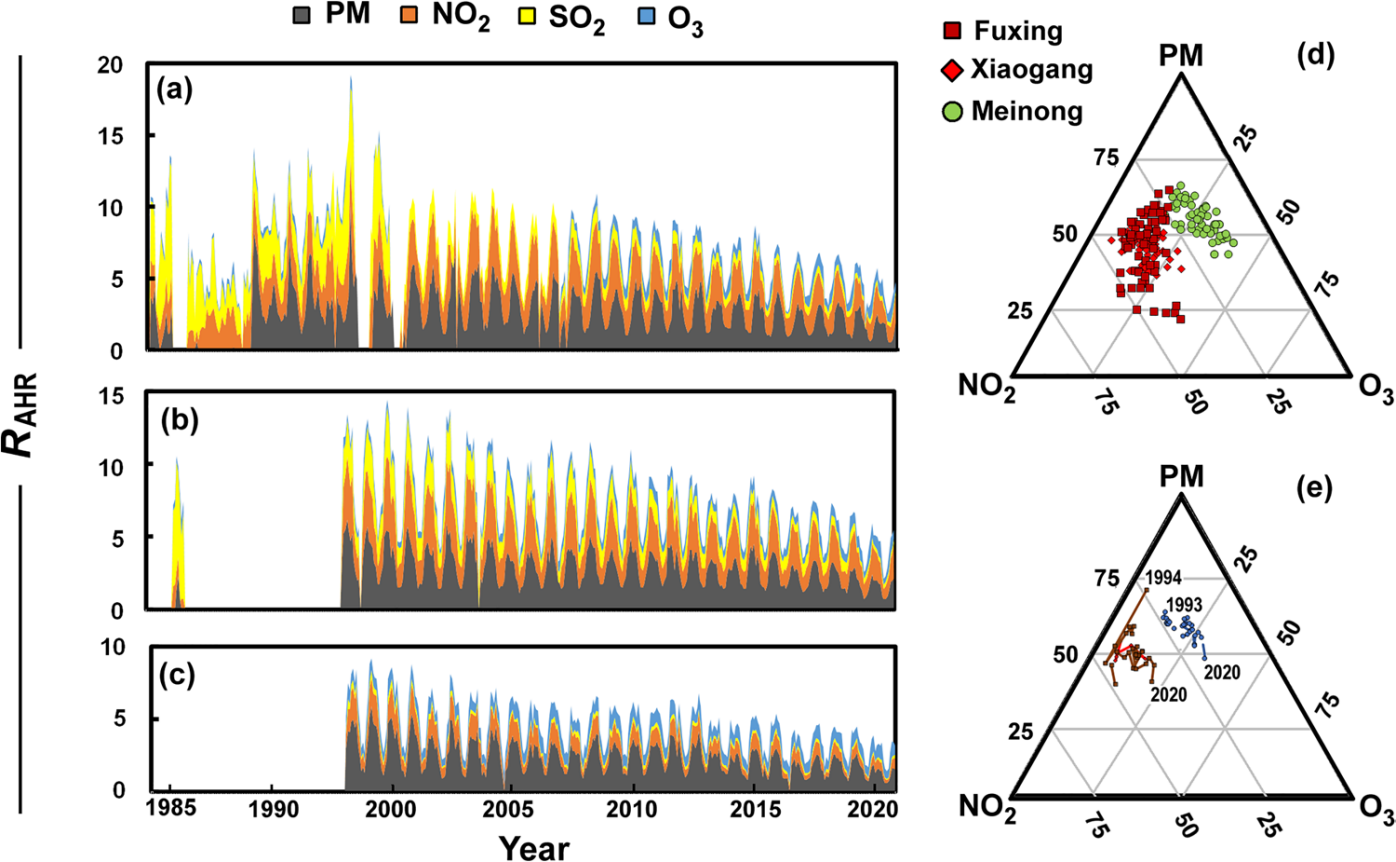
Monthly added contributions to health risk from PM_10_, NO_2_, SO_2_, and O_3_ at (**a**) Fuxing, (**b**) Xiaogang, and (**c**) Meinong. (**d**) Ternary diagram showing monthly added risk PM_10_, NO_2_, SO_2_, and O_3_ at Fuxing (red-brown squares), Xiaogang (red diamonds), and Meinong (green circles) from 2016 to 2020. (**e**) Added average annual contributions to health risk from PM_10_, NO_2_, SO_2_, and O_3_ at Fuxing (red-brown line), Xiaogang (few points as diamonds), and Meinong (green line)

Figure 4 shows the added daily health risk averaged for each month at (a) Fuxing, (b) Xiaogang, and (c) Meinong. The risk is much higher at the urban site in Xiaogang, but declines over time, in much the same way as the risk in rural Meinong. The risk from PM_10_ is relatively constant across the sites reflecting the broad distribution that arises from a multiplicity of sources. The balance of risk arises differently at the sites, so at Meinong a larger proportion comes from O_3_, while the effect of SO_2_ on health risk is very much lower compared with Fuxing, especially in the earlier years at this site. The proportions are clearer in the ternary plot of Fig. 4(d), which shows monthly risk across the 5-year period 2016–2020. It reveals the contemporary situation where the urban sites of Fuxing and Xiaogang are distinct from that in Meinong. The time trends for the relative risk over years 1993–2020 are shown in the ternary diagram of Fig. 4(e). This illustrates the transition to lower risk from particulate matter, but a greater proportion of risk that arises from O_3_, especially at the rural site, but the change has also been evident in the urban areas. Secondary pollutants are more difficult to control, separated as they are from their sources through a mediating chemistry. This difficulty was recognised with the discovery of photochemical smog 70 years ago (Brimblecombe 2014), and stresses a continued need for management of air quality that can address secondary pollutants.

### Agriculture

Agricultural crops are sensitive to O_3_ (Fuhrer et al. 1997), so this places pressure on food security (Wang et al. 2017). The hinterland to Kaohsiung is important in the production of a range of crops (ABKMG 2021): fruit (banana 57 900 t a^−1^, guava 71 468 t a^−1^, and pineapple 56 971 t a^−1^) and vegetables (bamboo shoots 20 497 t a^−1^, green soybeans 19 665 t a^−1^, tomatoes 12 902 t a^−1^, and radishes 11 496 t a^−1^).

**Fig. 5:**
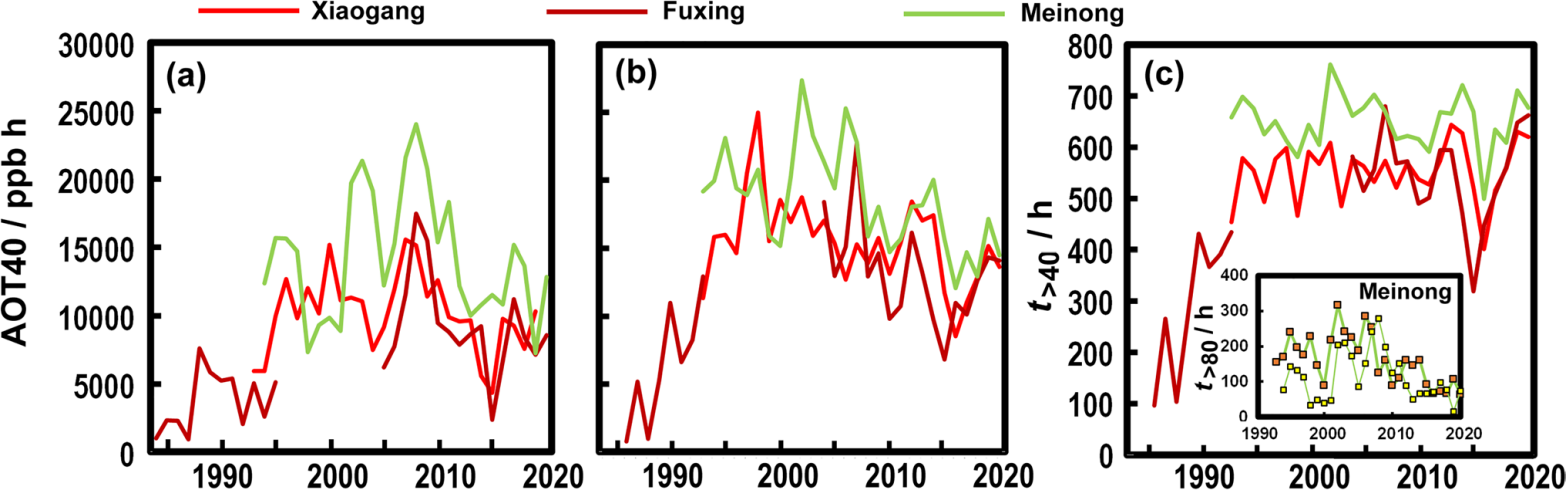
(**a**) AOT40 for the season March-May at Xiaogang, Fuxing, and Meinong. (**b**) AOT40 for the season September-November at the three sites. (**c**) Number of hours each year when O_3_ exceeded 40 ppb for the September-November season at Xiaogang, Fuxing, and Meinong. Inset shows the number of hours when O_3_ exceeded 80 ppb at Meinong for the March-May (small dots and fine line) and September-November (large squares and thick line) seasons

As crops accumulate damage over time, it is common to express risk to vegetation as AOT40 (Accumulated Ozone exposure over a threshold of 40 ppb during the day as ppb h). Summation is usually made over daylight hours during the crop’s growing season, although it is sometimes reported for each month. In Europe, the target value is 9000 ppb h considered over 5 years. The long-term objective is 3000 ppb h. In Europe, the growing season is typically May to July. Since Taiwan has a tropical climate, the growing season is more difficult to define because crops are grown all year round. Summer days are also not particularly long, so daylight hours are considered shorter in our calculations: 07:00 to 17:00. There are two periods (Fig. 2(i)) of high O_3_ levels, March to May and September to November (Chen et al. 2004). The 3-month long AOT40 for the two urban sites and the rural site at Meinong is shown in Fig. 5(a, b) for each O_3_ season. The mixing ratios of O_3_ increased over the late parts of the twentieth century, but it is somewhat uncertain because the records are difficult to overlap for cross-checking. We can see that Meinong is typically the highest, and the Wilcoxon signed-rank test shows it to be higher (*p*<.0001) than both Xiaogang (1994 to 2020) and Fuxing (2004 to 2020). Despite increases in O_3_ in general in the Kaohsiung area (Fig. 2(i)), there are hints the AOT40 is in decline particularly late in the year. The number of hours each year where late season O_3_ exceeds 40 ppb is plotted in Fig. 5(c), which suggests that although AOT40 might be decreasing, the number of hours above 40 ppb is relatively stable over recent years. The decline in AOT40 is mostly caused by a decrease in hours with high O_3_ (i.e. >80 ppb). These have declined recently especially in the late part of the year, but since 2001, in both ozone seasons, high O_3_ periods have become less common.

There are only a few studies of O_3_ and crop damage in Taiwan (e.g. Sheu and Liu 2003). In addition, there are few studies on major crops found in the Kaohsiung region, except for some studies on soybeans and tomatoes. Soybeans show visual damage after taking up several thousand ppb h over the entire growing season (Gosselin et al. 2020), conditions that were exceeded at the Meinong site (Fig. 5(a, b)). Over a period of weeks, tomatoes (*Lycopersicon esculentum* Mill. H-11) exposed to O_3_ at 200 and 350 ppb for 2.5 h at 3 days a week showed extensive foliar injury, defoliation, and reduction in biomass, although fruit yield was only lower at the higher mixing ratio (Oshima et al. 1975). However, such high values are not experienced at Meinong, and even an hourly mixing ratio >150 ppb is found less than 40 times since 1993. Nevertheless, most years exceed the European guideline value of 9000 ppb h, especially during the September to November period. The long-term objective 3000 ppb h is always exceeded. However, the experiments of Reinert et al. (1997) show that the Tiny Tim cherry tomato (*L. esculentum L.* cv. Tiny Tim) shows a 20% reduction in vegetative dry weight after 13 weeks exposure to just 80 ppb.

In Kaohsiung and the surrounding region, concentrations of O_3_ have remained high for the last quarter century. This represents both a risk to health and a threat to agriculture, so should be a matter of continued regulatory concern.

### Visibility

**Fig. 6:**
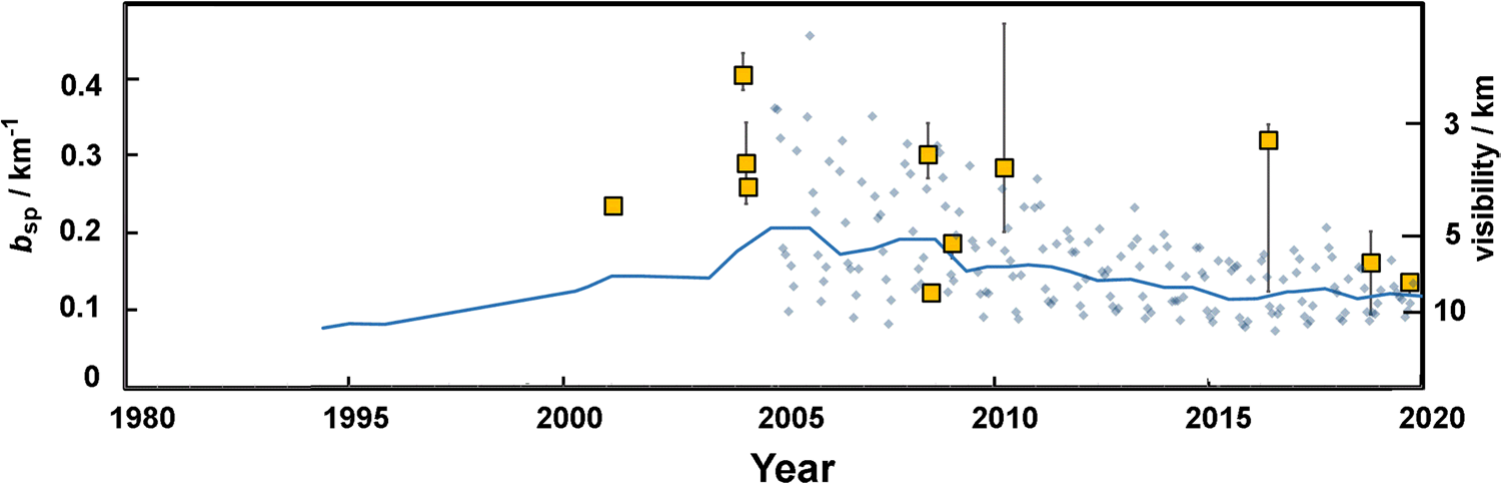
The light scattering coefficient (inverse visibility) in Kaohsiung with a line showing annual values from Maurer et al. (2019) extended to the present, and small shaded squares monthly values from CODiS. The large open squares are medians and the interquartile range calculated from the aerosol measurements using Eq. (2) Note: see supplement for details on the data sources. In calculations data from Chen et al. (2001), SO_4_^2−^ was set to 5 μg m^−3^ and Yang et al. (1998) PM_2.5_ was set to 30 μg m^−3^

Visibility is an important issue in the region. It is a publicly perceptible marker of changes in air pollution over long periods (Brimblecombe 2021), but studies from southern Taiwan do not always use the most recent data (Lee and Lai 2018). Maurer et al. (2019) were able to use the record up to 2016, which hints at the influence of PM_10_ on visibility and supports the notion that it has generally improved in Taiwan over recent decades. Yuan et al. (2006) suggest an empirical equation for the light scattering coefficient, *b*_sp_/km^−1^ as:

2 $${b}_{\mathrm{sp}}=0.0046{c}_{\left(\mathrm{NH}\right)42\mathrm{SO}4}+0.0067{c}_{\left(\mathrm{NH}\right)4\mathrm{NO}3}+0.0033{c}_{\mathrm{TC}}+{0.0032}_{\left({C}_{\mathrm{PM}2.5}-C0\right)}$$

where *c* is the concentration of aerosol components and *c*_0_ a remainder term. Yang et al. (2005) determined the amount of (NH_4_)_2_SO_4_ and NH_4_NO_3_ assuming that all SO_4_^2−^ and NO_3_^−^ was present as the ammonium salt, which requires that NH_4_^+^ be in excess, a reasonable assumption in Kaohsiung, and increasingly so given the decline in SO_2_ and NO_x_. However, as Li et al. (2022b) show for Beijing, the secondary aerosol can make a contribution to visibility that can outweigh the effects of primary emissions. The calculated visibility from aerosol measurements listed in the supplementary materials can be compared with observed visibility in Kaohsiung (Fig. 6), although such improvement might not be large enough to be obvious to the general population.

## Conclusion

This study has revealed long-term reductions in air pollution as a city transformed from an industrial base to a broader economy, with industry increasingly located around sites such as Xiaogang. On a day-to-day basis, mixing ratios of precursors and secondary pollutants might not correlate well, but it is likely that the effects of pollution chemistry and meteorology are smoothed out over the years, so primary and secondary pollutants seem to follow similar patterns. However, the response is non-linear, so the reduction in secondary SO_4_^2−^ in Kaohsiung has not been as large as the reduction in SO_2_ over the last decade. In parallel, the N/S ratio has increased with the decline in sulphur emissions. Air pollution concentrations in Kaohsiung have declined to a greater extent than the reduction in emissions. Both regulations and economic changes have enabled improvements in air quality in recent decades, yet O_3_ remains a problem. This secondary pollutant is difficult to control as it requires careful consideration of the balance of NO_x_ and hydrocarbons, especially as the NMHC emissions are no longer in sharp decline.

Future work could compare primary and secondary pollutant concentrations with emissions via modelling, although it may be difficult to collect data for spatially resolved emissions over long time periods. However, economic records could reveal fuel imports and farming statistics which would suggest the magnitude and distribution of emissions. Changes in secondary organic compounds were neglected in this study, although this would be an interesting topic for further research. Evaluating the impact of an emission reduction on change in visibility or health effects is important for formulating regulatory policy, and while modelling is available to link emissions to concentrations, nonlinear effects on exposure or health outcomes can be more difficult to represent.

## Supplementary Information

ESM 1 (DOCX 228 kb)

## Data Availability

The data is publicly available as denoted by URLs in the text.

## References

[CR1] ABKMG (2021) Agricultural Production (title in Chinese), Agricultural Bureau of Kaohsiung Municipal Government. https://agri.kcg.gov.tw/AgriculturaService/Agriculture/Produce.htm. Accessed 2 July 2022

[CR2] Alastuey A, Querol X, Rodriguez S, Plana F, Lopez-Soler A, Ruiz C, Mantilla E (2004). Monitoring of atmospheric particulate matter around sources of secondary inorganic aerosol. Atmos Environ.

[CR3] BP (2021). BP’s Statistical Review of World Energy 2021.

[CR4] Brimblecombe P (1977). London air pollution, 1500–1900. Atmos Environ.

[CR5] Brimblecombe P (1982). Trends in the deposition of sulphate and total solids in London. Sci Total Environ.

[CR6] Brimblecombe P (2005). The globalization of local air pollution. Globalizations.

[CR7] Brimblecombe P (2006). The Clean Air Act after 50 years. Weather.

[CR8] Brimblecombe P (2014). Deciphering the chemistry of Los Angeles smog, 1945–1995. In T*oxic Airs: Body, Place, Planet in Historical Perspective*.

[CR9] Brimblecombe P (2021). Visibility driven perception and regulation of air pollution in Hong Kong, 1968–2020. Environments.

[CR10] Brimblecombe P (2022). Trends in secondary inorganic particles in Hong Kong, 1995–2020. Atmos Environ.

[CR11] Chang CC, Sree U, Lin YS, Lo JG (2005). An examination of 7: 00–9: 00 PM ambient air volatile organics in different seasons of Kaohsiung city, southern Taiwan. Atmos Environ.

[CR12] Chen KS, Lin CF, Chou YM (2001). Determination of source contributions to ambient PM2. 5 in Kaohsiung, Taiwan, using a receptor model. J Air Waste Manage Assoc.

[CR13] Chen KS, Ho YT, Lai CH, Tsai YA, Chen SJ (2004). Trends in concentration of ground-level ozone and meteorological conditions during high ozone episodes in the Kao-Ping Airshed, Taiwan. J Air Waste Manag Assoc.

[CR14] Chen SP, Chang CC, Liu JJ, Chou CCK, Chang JS, Wang JL (2014). Recent improvement in air quality as evidenced by the island-wide monitoring network in Taiwan. Atmos Environ.

[CR15] Cheng B, Wang-Li L (2019). Responses of secondary inorganic PM2. 5 to precursor gases in an ammonia abundant area in North Carolina. Aerosol Air Qual Res.

[CR16] Cheng WH, Chou MS, Tung SC (2011). Gaseous ammonia emission from poultry facilities in Taiwan. Environ Eng Sci.

[CR17] Chow JC, Watson JG, Chaung CY (1983). Air pollution in the Republic of China (Taiwan). J Air Pollut Control Assoc.

[CR18] Davidson CI (1979). Air pollution in Pittsburgh: a historical perspective. J Air Pollut Control Assoc.

[CR19] DHR (2021) Dept. of Household Registration. Ministry of the Interior, Taiwan. https://www.ris.gov.tw/app/en. Accessed 2 July 2022

[CR20] Elsom DM (1992). Atmospheric pollution: a global problem.

[CR21] Everington K (2018) Taiwan to ban gasoline-powered scooters in 2035, Taiwan News (2018/11/05). https://www.taiwannews.com.tw/en/news/3568172. Accessed 2 July 2022

[CR22] Fuhrer J, Skärby L, Ashmore MR (1997). Critical levels for ozone effects on vegetation in Europe. Environ Pollut.

[CR23] Galindo N, Yubero E, Clemente Á, Nicolás JF, Varea M, Crespo J (2020). PM events and changes in the chemical composition of urban aerosols: a case study in the western Mediterranean. Chemosphere.

[CR24] Gosselin N, Sagan V, Maimaitiyiming M, Fishman J, Belina K, Podleski A, Maimaitijiang M, Bashir A, Balakrishna J, Dixon A (2020). Using visual ozone damage scores and spectroscopy to quantify soybean responses to background ozone. Remote Sens.

[CR25] Hsieh LT, Chen TC (2010). Characteristics of ambient ammonia levels measured in three different industrial parks in southern Taiwan. Aerosol Air Qual Res.

[CR26] Hsu CP, Wei VH (1971) Air pollution control in Taiwan Province (internal report Health Department, Taipei)

[CR27] Hung-Lung C, Jiun-Horng T, Shih-Yu C, Kuo-Hsiung L, Sen-Yi M (2007). VOC concentration profiles in an ozone non-attainment area: a case study in an urban and industrial complex metroplex in southern Taiwan. Atmos Environ.

[CR28] Ishikawa Y, Hara H (1997). Historical change in precipitation pH at Kobe, Japan: 1935–1961. Atmos Environ.

[CR29] Kawamura K, Steinberg S, Ng L, Kaplan IR (2001). Wet deposition of low molecular weight mono-and di-carboxylic acids, aldehydes and inorganic species in Los Angeles. Atmos Environ.

[CR30] KCG (2010) The current situation and challenges of the development of Kaohsiung's cities (in Chinese). https://orgws.kcg.gov.tw/001/KcgOrgUploadFiles/334/relfile/0/69818/33338bf1-b27e-4a50-b5dd-c937bff20905.pdf. Accessed 24 Dec 2021

[CR31] KCG (2021) Survey of Budget Department of Budget, Kaohsiung City Government, Accounting and Statistics. https://bas.kcg.gov.tw/. Accessed 2 July 2022

[CR32] KCGDG (2021) Statistical Information Network of Kaohsiung City, Kaohsiung City Government. https://kcgdg.kcg.gov.tw/kcgstat/page/default.aspx. Accessed 22 July 2021

[CR33] KEC (2021) Economy, industries & development, Kaohsiung Exhibition Center. http://www.kecc.com.tw/cityEconomy.asp. Accessed 22 Dec 2021

[CR34] Kuo YM, Chiu CH, Yu HL (2015). Influences of ambient air pollutants and meteorological conditions on ozone variations in Kaohsiung, Taiwan. Stoch Env Res Risk A.

[CR35] Lai YC, Tsai CH, Chen YL, Chang-Chien GP (2017). Distribution and sources of atmospheric polycyclic aromatic hydrocarbons at an industrial region in Kaohsiung, Taiwan. Aerosol Air Qual Res.

[CR36] Lee CG (2006). Study of the effect of aerosol characteristics and meteorological parameters on visibility in Urban Kaohsiung. Thesis, Environmental Engineering, National Sun-Yat Sen University.

[CR37] Lee CG, Lai WL (2018). Effects of different factors on the visibility in Kaohsiung Area using hierarchical regression. In International Conference on Genetic and Evolutionary Computing.

[CR38] Lee CG, Yuan CS, Chang JC, Yuan C (2005). Effects of aerosol species on atmospheric visibility in Kaohsiung city, Taiwan. J Air Waste Manage Assoc.

[CR39] Lee CS, Chang KH, Kim H (2018). Long-term (2005–2015) trend analysis of PM 2.5 precursor gas NO2 and SO2 concentrations in Taiwan. Environ Sci Pollut Res.

[CR40] Li XB, Yuan B, Parrish DD, Chen D, Song Y, Yang S, Liu Z, Shao M (2022). Long-term trend of ozone in southern China reveals future mitigation strategy for air pollution. Atmos Environ.

[CR41] Li Z, Sun Y, Wang Q, Xin J, Sun J, Lei L, Li J, Fu P, Wang Z (2022). Nitrate and secondary organic aerosol dominated particle light extinction in Beijing due to clean air action. Atmos Environ.

[CR42] Liang WJ, Lee CT (1980). The optimization evaluation of SO2 air pollution and monitor network in Kaohsiung area. J Chin Inst Eng.

[CR43] Lin JJ (2002). Characterization of the major chemical species in PM2. 5 in the Kaohsiung City, Taiwan. Atmos Environ.

[CR44] Liu T, Wang X, Wang B, Ding X, Deng W, Lü S, Zhang Y (2014). Emission factor of ammonia (NH3) from on-road vehicles in China: tunnel tests in urban Guangzhou. Environ Res Lett.

[CR45] Maurer M, Klemm O, Lokys HL, Lin NH (2019). Trends of fog and visibility in Taiwan: climate change or air quality improvement?. Aerosol Air Qual Res.

[CR46] Oshima RJ, Taylor OC, Braegelmann PK, Baldwin DW (1975). Effect of ozone on the yield and plant biomass of a commercial variety of tomato. Am Soc Agron Crop Sci Soc Am Soil Sci Soc Am.

[CR47] Power A, Worsley A, Charlesworth SM, Colin A (2018). Historical urban pollution. Booth Urban Pollution: Science and Management.

[CR48] Ravishankara AR (1997). Heterogeneous and multiphase chemistry in the troposphere. Science.

[CR49] Reinert RA, Eason G, Barton J (1997). Growth and fruiting of tomato as influenced by elevated carbon dioxide and ozone. New Phytol.

[CR50] Selya RM (1975). Water and air pollution in Taiwan. J Dev Areas.

[CR51] Shaw D, Hung MF (2001). Evolution and evaluation of air pollution control policy in Taiwan. Environ Econ Policy Stud.

[CR52] Shen H, Cheng PH, Yuan CS, Yang ZM, Ie IR (2020). Chemical characteristics, spatiotemporal distribution, and source apportionment of PM2. 5 surrounding industrial complexes in Southern Kaohsiung. Aerosol Air Qual Res.

[CR53] Sheu BH, Liu CP (2003) Air pollution impacts on vegetation in Taiwan. Air Pollut Impacts Crops For–a Global Assess:145–163

[CR54] Shiu CJ, Liu SC, Chang CC, Chen JP, Chou CC, Lin CY, Young CY (2007). Photochemical production of ozone and control strategy for Southern Taiwan. Atmos Environ.

[CR55] Smit R, Ntziachristos L, Boulter P (2010). Validation of road vehicle and traffic emission models–a review and meta-analysis. Atmos Environ.

[CR56] Tang DTC (1993). The environmental laws and policies of Taiwan: A comparative law perspective. Pac Rim Law Policy J.

[CR57] Tang CP, Tang SY (2000) Democratizing bureaucracy: the political economy of environmental impact assessment and air pollution prevention fees in Taiwan. Comp Polit:81–99

[CR58] Tsai PL (1999). Explaining Taiwan's economic miracle: are the revisionists right?. Agenda: J Policy Anal Reform.

[CR59] Tsai HH, Liu YF, Yuan CS, Chen WH, Lin YC, Hung CH, Jen YH, Ie IR, Yang HY (2012). Vertical profile and spatial distribution of ozone and its precursors at the inland and offshore of an industrial city. Aerosol Air Qual Res.

[CR60] Tsai JH, Chang LP, Chiang HL (2013). Size mass distribution of water-soluble ionic species and gas conversion to sulfate and nitrate in particulate matter in southern Taiwan. Environ Sci Pollut Res.

[CR61] Vannest KJ, Parker RI, Gonen O, Adiguzel T (2016) Single case research: web based calculators for SCR analysis. (Version 2.0) [Web-based application]. College station: Texas A&M University. http://www.singlecaseresearch.org/calculators/theil-sen. Accessed 29 June 2021

[CR62] Wang T, Xue L, Brimblecombe P, Lam YF, Li L, Zhang L (2017). Ozone pollution in China: A review of concentrations, meteorological influences, chemical precursors, and effects. Sci Total Environ.

[CR63] Wei W-H (1966). Air pollution control on Taiwan.

[CR64] Wong TW, Tam WWS, Lau AKH, Ng SKW, Yu ITS, Wong AHS, Yeung DA (2012) Study of the air pollution index reporting system, report to the air services group; Tender Ref. AP 07-085. The Environmental Protection Department of HKSAR, Kowloon

[CR65] Yang CY, Wang JD, Chan CC, Hwang JS, Chen PC (1998). Respiratory symptoms of primary school children living in a petrochemical polluted area in Taiwan. Pediatr Pulmonol.

[CR66] Yang H, Yu JZ, Ho SSH, Xu J, Wu WS, Wan CH, Wang X, Wang X, Wang L (2005). The chemical composition of inorganic and carbonaceous materials in PM2. 5 in Nanjing, China. Atmos Environ.

[CR67] Yuan CS, Lee CG, Liu SH, Chang JC, Yuan C, Yang HY (2006). Correlation of atmospheric visibility with chemical composition of Kaohsiung aerosols. Atmos Res.

[CR68] Zheng JY, Yin SS, Kang DW, Che WW, Zhong LJ (2012). Development and uncertainty analysis of a high-resolution NH_3_ emissions inventory and its implications with precipitation over the Pearl River Delta region, China. Atmos Chem Physics.

