# Supplementary material for: Fifty-year change in air pollution in Kaohsiung, Taiwan
Source: Environ Sci Pollut Res Int. 2022 Jul 4;29(56):84521–31. doi: 10.1007/s11356-022-21756-z (PMC9646597; doi:10.1007/s11356-022-21756-z)
Supplement: Supplementary file 1 — (DOCX 228 kb) [file 11356_2022_21756_MOESM1_ESM.docx]

**SUPPLEMENTARY TABLE**

Table S1 Sites and sampling durations from studies used in this research, with additional detail as footnotes. The site code letters relate to those plotted on the map in Fig. S1. Spellings of place names adopted by references are listed along with the transliteration adopted here and their Chinese names.

| **Authors** | **Site** | **EPA site used transliterated and Chinese** | **Sampler** | | **flow rate L min^-1^** | **Sampling season** | **Sampling date (ISO 8601)** |
| --- | --- | --- | --- | --- | --- | --- | --- |
| **1. Yang et al., 1998** | Linyuan | Linyuan (林園) | Beta attenuation | | | winter, spring | 1994-10/1995-4 |
| **2. Lin, 2002** | Nan-tze | Nanzih (楠梓) | Graseby Andersen G241 | | 16.7 | winter, spring | 1998-11-11/12, 1998-12-28/30, 1999-02-27/28, 1999-04-24/25 |
|  | Tso-ying | Zuoying (左營) |  |  |  |  | 1998-11-14/15, 1999-01-06/08,   1999-03-01/02, 1999-04-26/27 |
|  | Chien-chin | Qianjin (前金) |  |  |  |  | 1998-12-10/11, 1999-02-22/23, 1999-03-21/22 |
|  | San-min | Sanmin (三民) |  |  |  |  | 1998-12-08/09, 1999-01-22/24, 1999-02-05/11, 1999-04-13/14 |
|  | Hsiao-kang | Xiaogang (小港) |  |  |  |  | 1998-12-15/17, 1999-01-31, 1999-02-01, 1999-04-08/09 |
|  | Chien-chen | Qianzhen (前鎮) |  |  |  |  | 1998-11-23/24, 1998-12-05/06, 1999-01-10/12, 1999-02-25/26, 1999-04-15/16 |
| **3. Lee et al., 2005** | Chentserng | Qianji (前金) | Anderson Model 241 | | 16.7 | winter, spring | 1999-01-28/1999-02-06  and 1999-05-21/1999-05-30 |
| **4. Chen et al., 2001** | Tzuoying | Zuoying (左營) | Graseby Andersen 246b | | 16.67 | spring | 1999-2-24/1999-3-3 |
|  | Chianjen | Qianji (前金) |  |  |  |  | 1999-2-24/1999-3-3 |
|  | Shiugang | Xiaogang (小港) |  |  |  |  | 1999-2-24/1999-3-3 |
| **5. Hsu, 2008** | Daliao | Daliao (大寮) | Graseby Anderson G241 | | 16.7 | winter | 2004-10-22/2004-11-03 |
|  | Tzuoying | Zuoying (左營) |  |  |  |  | 2004-10-22/2004-11-03 |
| **6. Chang et al., 2011** | Daliao | Daliao (大寮) | University Research Glassware; URG, Chapel Hill Inc., USA | | 16.7 | winter | 2004-10-22/2004-11-03 |
|  | Tzouying | Zuoying (左營) |  |  |  |  | 2004-10-22/2004-11-03 |
| **7. Kuo et al., 2011** | Foo-yin (Fouyin) | Daliao (大寮) | Thermo Electron Co., East Greenbush, NY, USA | | | summer | 2005-5/2005-8 |
|  | Cian-jhen | Qianji (前金) |  |  |  |  | 2005-5/2005-8 |
|  | Chiau-tou | Qiaotou (橋頭) |  |  |  |  | 2005-5/2005-8 |
| **8. Tsai et al., 2011** | NKU | Qiaotou (橋頭)/ Nanzih (楠梓)/ Renwu (仁武)/ Zuoying (左營) | Graseby Anderson 241 | 16.7 | | summer, winter, spring | 2006-08-16/18,  2006-11-02/04 and 2007-05-02/04 |
|  | FIC | Daliao (大寮)/ Fengshan (鳳山)/ Xiaogang (小港) |  |  |  |  | 2006-08-16/18,  2006-11-02/04 and 2007-05-02/04 |
|  | HLC | Linyuan (林園) |  |  |  |  | 2006-08-16/18,  2006-11-02/04 and 2007-05-02/04 |
|  | OSB | Linyuan (林園)/ Xiaogang (小港) |  |  |  |  | 2006-08-16/18,  2006-11-02/04 and 2007-05-02/04 , 2007-01-24/26 and 2007-03-06/08 |
|  | ZIJ | Zuoying (左營) |  |  |  |  | 2006-08-16/18,  2006-11-02/04 and 2007-05-02/04 , 2007-01-24/26 and 2007-03-06/08 |
|  | CSJ | Qianjin (前金) |  |  |  |  | 2006-08-16/18,  2006-11-02/04 and 2007-05-02/04 , 2007-01-24/26 and 2007-03-06/08 |
|  | NKFU | Qiaotou (橋頭)/ Nanzih (楠梓) |  |  |  |  | 2006-08-16/18,  2006-11-02/04 and 2007-05-02/04 , 2007-01-24/26 and 2007-03-06/08 |
| **9. Yang et al., 2017** | Chien-Chin(Chien-Chin Primary School, QPS) | Qianjin (前金) | BGI PQ200 | 16.7 | | winter, spring | 2014-12/2015-05 |
|  | Siao-Gang(Siao-Gang Senior High School, XHS) | Xiaogang (小港) |  |  |  |  | 2014-12/2015-05 |
| **10. Shen et al., 2020** | NOU | Xiaogang (小港) | BGI PQ200 | 16.67 | | annual | 2017-12-04/08,  2018-03-12/16, 2018-07-10/13 and 2018-11-17/21 |
|  | FY | Daliao (大寮) |  |  |  |  | 2017-12-04/08,  2018-03-12/16, 2018-07-10/13 and 2018-11-17/21 |
|  | FL | Xiaogang (小港)/ Linyuan (林園) |  |  |  |  | 2017-12-04/08,  2018-03-12/16, 2018-07-10/13 and 2018-11-17/21 |

**Footnotes**

1. The monitoring stations were fully automatic. The air pollution data (e.g. SO_2_, NO_2_, PM_10_) were retrieved from the air quality monitoring data of the EPA for the whole of 1994. SO_2_ was measured by means of a UV fluorescence instrument, NO_2_ by a chemiluminescent apparatus, and PM_10_ by a beta attenuation method. We also used the Harvard-EPA annular denudersystems,12,13 to collect acidic aerosols. The samplers were placed beside the EPA monitoring station. Acidic aerosols were monitored 1 day per 2 weeks from 1994-10/1995-04. During each sampling day, one 24-hour sample was taken.
2. Ambient particle concentrations were measured using a dichotomous sampler (Graseby Andersen G241). This dichotomous sampler was equipped with an inlet having a 10-mm cut-point. The particles below 10 mm aerodynamic diameter (PM_10_) were divided into two size fractions using a virtual impactor with a 2.5-mm cut-point when entering the sampler. These two fractions are classified as a coarse fraction (2.5 mmodiametero10 mm, PM_2.5–10_) and a fine fraction (diameter o2.5 mm, PM_2.5_). The dichotomous sampler was operated at a total flow rate of 16.7 l min^-1^ (1.67 and 15 l min^-1^ for coarse and fine flows). Particles were collected using 37-mm quartz fiber filters (Pallflex 2500 QAT-UP, 37 mm) supported by polyolefin rings. To reduce the carbonaceous species background level in the filter, filters were pre-heated before sampling at 900℃ for 90 min and then placed in clean polyethylene Petri dishes. The Petri dishes were then wrapped with Teflon tape and aluminum foil, and stored in a freezer until field measurement. Before and after field sampling, the filters were weighed on an electronic balance (Mettler Toledo AT261) with a reading precision of 10 mg to determine the mass concentration after having been conditioned at 25℃ and 40% relative humidity for 24 h. After collection from the field, sample filters were stored in a refrigerator at 4℃ before chemical analysis to limit losses of volatile components (Chow, 1995). Chemical characteristics of major species in fine particles (PM_2.5_) were presented in this paper. Coarse (PM_2.5–10_) and fine (PM_2.5_) particle samples were used to discuss the differences between PM_2.5_ and PM_10_ for specific components in Lin and Tai (2001) and Lin (2002). The observations were carried out from November 1998 to April 1999 in Kaohsiung City.Samples were collected during periods of no rain. Concentrations of ambient particle samples were calculated as a 24-h average (~0700–0700 next day). Hourly wind speed was recorded and the average wind speed during each sample period was then calculated..
3. Aerosol particles were simultaneously sampled during intensive observation periods to ascertain the influence of aerosol characteristics on visibility impairment in urban area of Kaohsiung. Atmospheric aerosols were collected at Chentserng (i.e. Qianji) Air Quality Monitoring Station, on the 10–m-high roof of a 3-story building located in the central metropolitan Kaohsiung (Figure 1). Both fine and coarse particles (i.e. PM_2.5_ and PM_2.5–10_) were collected on the quartz fiber filters by a dichotomous sampler (Anderson Model 241) with a total flow rate of 16.7 l/min. The 37-mm diameter filters were initially conditioned at 23±2°C and 40%±5% relative humidity for 24 hr before sampling. Aerosol particles were collected for a consecutive 5-hr periods in both the morning (07:00–24:00) and afternoon (12:00-17:00) during the periods of intensive sampling.
4. Sampling concurrently at the three sites from 1999-2-24 to1999-3-3, so that spatial variations could be investigated. There were two sampling periods each day, that is, the day period ran from 7:00-19:00, and the night period ran from 19:00-07:00, each lasting exactly 12 hr. Three identical dichotomous samplers (Model 246b, Graseby 4Andersen Inc.) were used for sampling ambient PM_2.5_. The total air flow rate of the sampler was maintained at 16.67 L min^-1^ —15 L min^-1^ for PM_2.5-10_ and 1.67 L min^-1^ for PM_2.5_. Quartz filters of 37 mm diameter were used for intercepting the airborne particles.
5. Ambient PM was taken by a dichotomous sampler (Graseby Adenson G 241) equipped with an inlet of a 10μm cut point. PM less than 10μm in aerodynamic diameter (PM_10_) was divided into two size fractions when entering the sampler using a virtual impactor with a 2.5μm cut point. The two size fractions were classified as a coarse PM_10–2.5_ fraction and the PM_2.5_ fine fraction. Particulate mass concentration of PM_10_ is the sum of PM_2.5_ and PM_10–2.5_ in this study. The total flow rate of the dichotomous sampler was 16.7 L min^-1^. It was split into 1.67 and 15 L min^-1^ for coarse and fine flows, respectively. Twenty-six samples were taken for each cut size (PM_2.5_ and PM_10–2.5_), with a total of 104 samples for both cut size particles at the two stations.

Particulate matter was collected using 37 mm quartz fiber filters (Pallflex 2500 QAT-UP, 37 mm) supported by polyolefin rings. Filters were pretreated before sampling at 900°C for 2 hr to reduce the carbonaceous species background level and interference by other volatile species in the filter and reduce the artifact effect caused by the filter. The background concentration of ionic species in the filter was low.

1. The experiment was conducted in Kaohsiung in southern Taiwan during the period of 2004-10-22 to 2004-11-03. Two stations of the Taiwan Air Quality Monitoring Network

(which was established by the Taiwan Environmental Protection Agency in 1993), Daliao and Tzouying, were chosen as sampling sites for the experiment (Fig. 1). At each site, samples were collected during the periods of 07:00-19:00 and 19:00-07:00 the following day to enable characterization of air pollutants during the day and night, respectively.

The denuder system employed in this study was composed of a cyclone with a cut-off diameter of 2.5 μm (University Research Glassware; URG, Chapel Hill Inc., USA) followed by four annular denuders (URG-2000-30EH), a filter pack, a flow controller and a pump (USEPA, 1998). Airflow was set at a constant rate of 16.7 L min^−1^.

1. All gaseous pollutants and PM_2.5_ instruments were designated by the USEPA equivalent or reference methods. A collocated Rupprecht and Patashnick 1400a tapered element oscillating microbalance sampler (Thermo Electron Co., East Greenbush, NY, USA) was used to determine the concentrations of PM_2.5_ (EQPM-1090-079). A semi-continuous carbon aerosol analyzer (model 5040, Sunset Laboratory Inc., Portland, OR, USA) using a thermo-optical transmission (Schmid et al., 2001) was employed to determine the concentrations of organic and elemental carbons in PM_2.5_. An ambient particulate nitrate monitor (8400N, Rupprecht and Patashnick Co., Inc., Albany, NY, USA) and ambient particulate sulfate monitor (8400S, Rupprecht and Patashnick Co., Inc.) were used to determine the concentrations of nitrate and sulfate, respectively. Carbon monoxide concentrations were determined by the APMA-360CE gas filter correlation ambient CO analyzer (Horiba Instruments Inc., Irvine, CA, USA; RFCA-0895-160).
2. At these sampling sites, mobile air quality monitoring vehicles simultaneously collected atmospheric PM (PM_2.5_, PM_2.5-10_, and PM_10_) with dichotomous samplers (Anderson, Model Series 241). The sampling flow rate of the dichotomous sampler was 16.7 L min^−1^, and 37 mm quartz filter were used in this study.
3. Twenty-four hour sampling of PM_2.5_ was conducted simultaneously at the aforementioned three sampling sites from 08:00-08:00 of the sequential day. The sampler used for collecting PM_2.5_ in the atmosphere at each sampling site was BGI PQ200 with WINS impactor under an air flow rate of 16.7 L min^–1^. Before and after PM_2.5_ sampling, the quartz fiber filters of 47 mm diameter were conditioned in the desiccators at constant temperature (25 ± 3°C) and relative humidity (45 ± 5%) for at least 24 h (Yuan et al., 2004, 2006; Li et al., 2013a, b), and further weighted by an analytical microbalance (Sartorius MC 5) with a mass precision of 10^–6^ g.
4. Twelve-hour PM_2.5_ samples at a flow rate of 16.67 L min^–1^ from 2017-12 to 2018-9 in different seasons. Four PM_2.5_ sampling protocols were conducted on 2017-12-04/08 (winter samples), 2018-03-12/16 (spring samples), 2018-07-10/13 (summer samples), and 2018-11-17/21 (fall samples), respectively. Quartz fibrous filters of 47 mm were used in the PM_2.5_ samplers for collecting 12-h PM_2.5_ for consecutive five days in each season. Daytime PM_2.5_ sampling started from 07:00 to 19:00, while nighttime PM_2.5_ sampling started from 07:00 to 19:00 (next day). After sampling, PM_2.5_ filters were then transported back to Air Pollution Laboratory in the Institute of Environmental Engineering at National Sun Yat-Sen University as soon as possible and temporarily conditioned in a thermostatic chamber with a constant temperature (T) of 20–25°C and a relative humidity (RH) of 40 ± 5% for at least 48 h for weighing by an analytical microbalance with the precision of 1 μg (MSA6.6S, Sartorius) to determine the mass concentration of PM_2.5_.


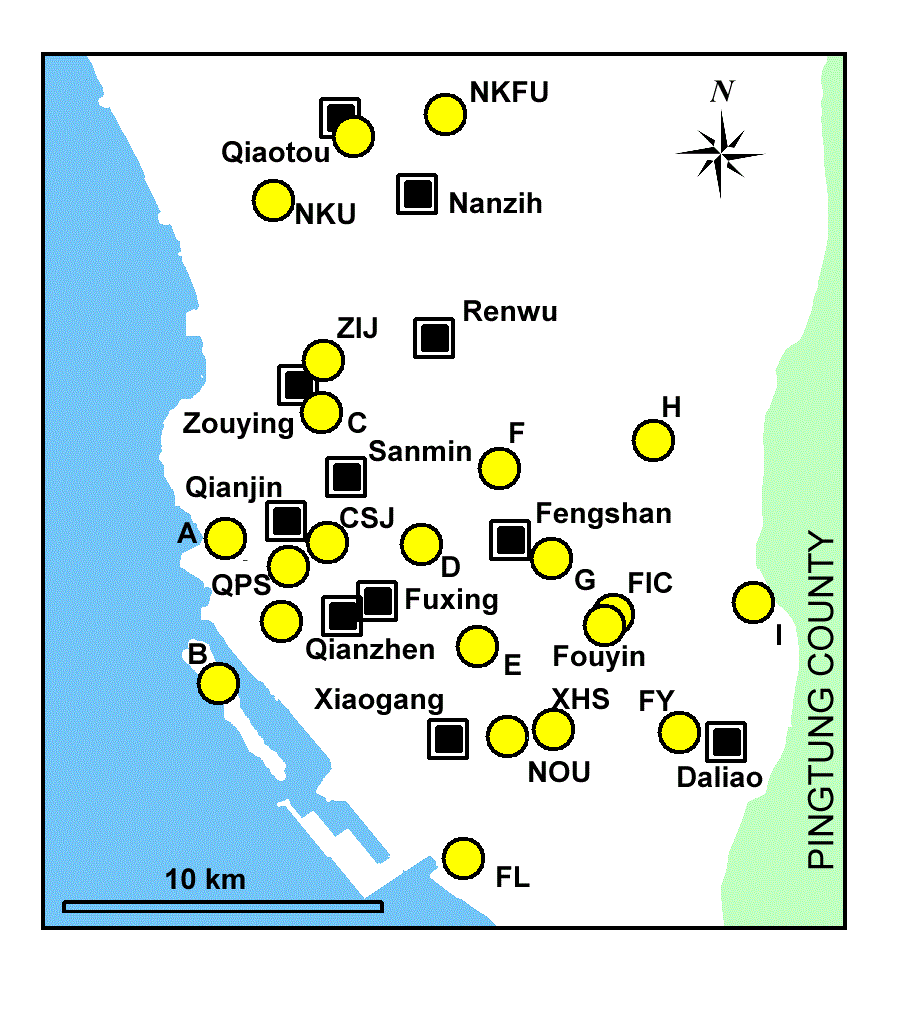


Fig. 1. Sampling sites (circles) and TEPA sites (squares). Notes: The site code letters defined in Table S1 along with transliteration of Chinese place names. Note: A-I are not used in this study.


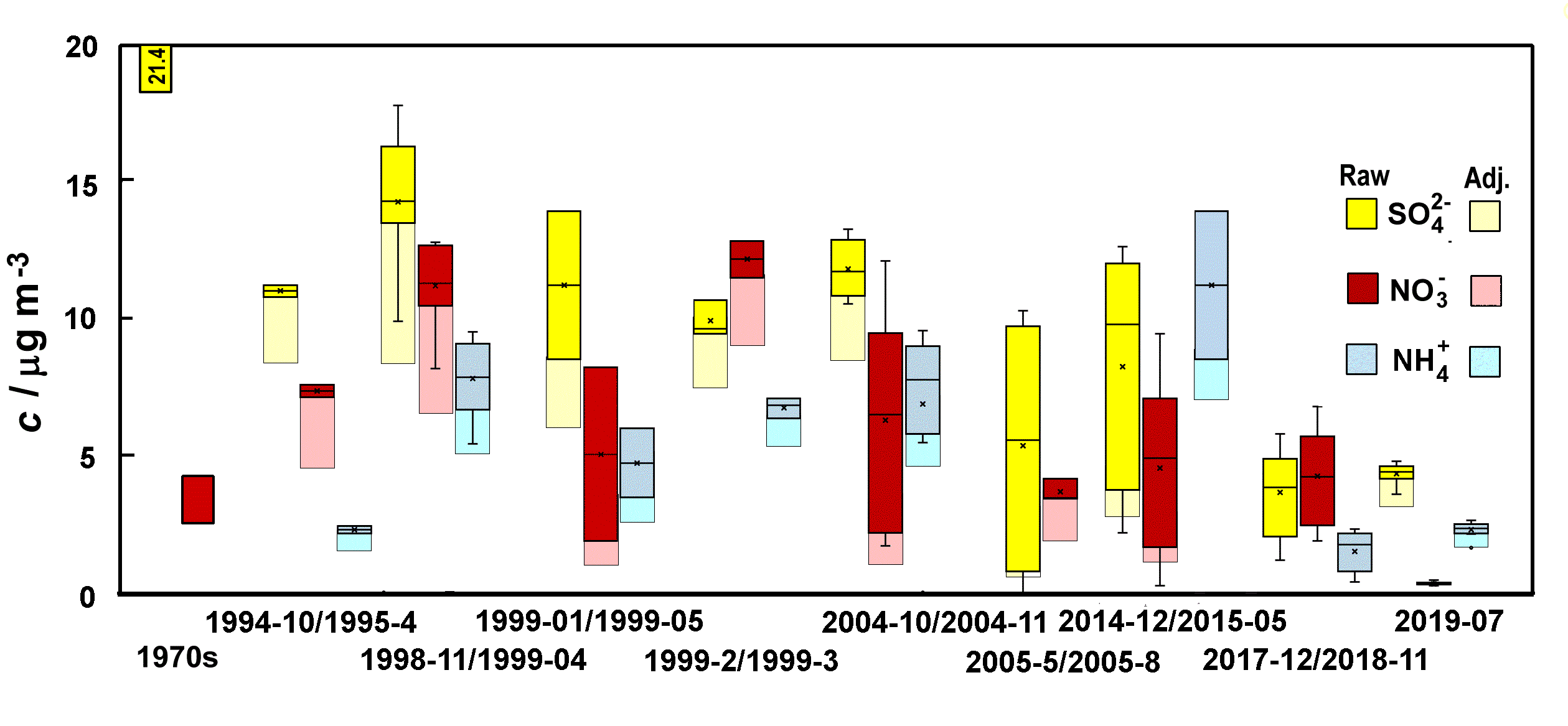


Fig. S2. Aerosol concentrations from studies made over more than twenty years (a) box and whisker plots for SO_4_^2-^, NO_3_^-^ and NH_4_^+^, as μg m^-3^ in chronological order of dates of campaigns, with lighter colours to suggested the likely downward adjustment (adj.) of the lower quartile from winter-spring to annual values.  The adjustments (adj.) to Fig. S2 were derived from the study of Shen et al (2018) suggested that seasonally SO_4_^2-^, NO_3_^-^ and NH_4_^+^ were high in winter high by 25%, 50% and 25%; spring by 25%, 25% and 25%; summer low as -50%, -50% and -50%; and autumn -25%, 0% and -25% respectively.

Although many of the campaigns took place in the polluted winter and spring seasons, adjustments (adj.) for this do not change the picture, which suggests notable decreases in SO_4_^2-^concentrations across the period and in more recent years probable decreases in the three inorganic aerosol components, suggesting better air quality and a lower risk to health and better visibility.
